# Effect of Inter-Domain Linker Composition on Biodistribution of ABD-Fused Affibody-Drug Conjugates Targeting HER2

**DOI:** 10.3390/pharmaceutics14030522

**Published:** 2022-02-26

**Authors:** Tianqi Xu, Jie Zhang, Maryam Oroujeni, Maria S. Tretyakova, Vitalina Bodenko, Mikhail V. Belousov, Anna Orlova, Vladimir Tolmachev, Anzhelika Vorobyeva, Torbjörn Gräslund

**Affiliations:** 1 Department of Immunology, Genetics and Pathology, Uppsala University, 751 85 Uppsala, Sweden; tianqi.xu@igp.uu.se (T.X.); maryam.oroujeni@igp.uu.se (M.O.); vladimir.tolmachev@igp.uu.se (V.T.); 2 Department of Protein Science, KTH Royal Institute of Technology, Roslagstullsbacken 21, 114 17 Stockholm, Sweden; jiezha@kth.se (J.Z.); torbjorn@kth.se (T.G.); 3 Department of Science and Development, Affibody AB, 171 65 Solna, Sweden; 4 Research Centrum for Oncotheranostics, Research School of Chemistry and Applied Biomedical Sciences, Tomsk Polytechnic University, 634050 Tomsk, Russia; trremar@mail.ru (M.S.T.); bodenkovitalina@gmail.com (V.B.); anna.orlova@ilk.uu.se (A.O.); 5 Cancer Research Institute, Tomsk National Research Medical Center, Russian Academy of Sciences, 634009 Tomsk, Russia; 6 Department of Pharmaceutical Analysis, Siberian State Medical University, Ministry of Health of the Russian Federation, 634050 Tomsk, Russia; mvb63@mail.ru; 7 Research School of Chemistry and Applied Biomedical Sciences, National Research Tomsk Polytechnic University, 634050 Tomsk, Russia; 8 Department of Medicinal Chemistry, Uppsala University, 751 23 Uppsala, Sweden

**Keywords:** affibody molecule, human epidermal growth factor receptor 2, HER2, SKOV3, emtansine, DM1, albumin binding domain, affibody drug conjugate, AffiDC

## Abstract

Targeted drug conjugates based on Affibody molecules fused to an albumin-binding domain (ABD) for half-life extension have demonstrated potent anti-tumor activity in preclinical therapeutic studies. Furthermore, optimization of their molecular design might increase the cytotoxic effect on tumors and minimize systemic toxicity. This study aimed to investigate the influence of length and composition of a linker between the human epidermal growth factor receptor 2 (HER2)-targeted affibody molecule (Z_HER2:2891_) and the ABD domain on functionality and biodistribution of affibody-drug conjugates containing a microtubulin inhibitor mertansin (mcDM1) (AffiDCs). Two conjugates, having a trimeric (S_3_G)_3_ linker or a trimeric (G_3_S)_3_ linker were produced, radiolabeled with ^99m^Tc(CO)_3_, and compared side-by-side in vitro and in vivo with the original Z_HER2:2891_-G_4_S-ABD-mcDM1 conjugate having a monomeric G_4_S linker. Both conjugates with longer linkers had a decreased affinity to HER2 and mouse and human serum albumin in vitro, however, no differences in blood retention were observed in NMRI mice up to 24 h post injection. The use of both (S_3_G)_3_ and (G_3_S)_3_ linkers reduced liver uptake of AffiDCs by approximately 1.2-fold compared with the use of a G_4_S linker. This finding provides important insights into the molecular design for the development of targeted drug conjugates with reduced hepatic uptake.

## 1. Introduction

The human epidermal growth factor receptor 2 (HER2) belongs to the human epidermal growth factor receptor (HER/EGFR/ERBB) family and is a transmembrane protein with tyrosine kinase activity [1]. HER2 is overexpressed in several types of cancer with its expression being low in normal adult tissues, which gives an opportunity for HER2-targeted therapy. Its overexpression and/or the amplification of its encoding gene, *ERBB2*, is also associated with poor response to chemotherapy and increased recurrence rate in breast cancer [2].

Targeted drug delivery using proteins coupled with cytotoxic agents is a promising way to treat disseminated cancer and has been intensively investigated in recent years. Antibody-drug conjugates (ADCs) based on a monoclonal antibody (mAb) for selective delivery of a drug to malignant tumors is the most common and well-studied format of targeted drugs [3]. Two HER2-targeting ADCs, trastuzumab emtansine (T-DM1) and trastuzumab deruxtecan, have been approved for clinical use by the U.S. Food and Drug Administration (FDA) for treatment of metastatic HER2-positive breast cancer. The use of these ADCs has provided a significant increase in progression-free survival of patients compared with standard treatment. However, the use of mAbs for targeted delivery is associated with several drawbacks. The large size of mAbs (150 kDa) limits their penetration into tumor tissue, reducing the efficiency of drug delivery to targeted cells [3,4,5]. Furthermore, many of the current methods for drug conjugation to antibodies provide heterogeneous mixtures with low control over the number of conjugations per antibody (drug-to-antibody ratio, DAR) and the spatial positioning of the drugs, resulting in a product consisting of species with variable pharmacokinetic properties, efficacy, and possible regulatory issues [6]. The development of strategies for site-specific conjugation of drugs to mAbs is being intensively researched [7].

Engineered scaffold proteins (ESPs) might serve as alternative carriers of cytotoxic drugs and could overcome the abovementioned problems of mAbs. An example of ESPs is the affibody molecules (6.5 kDa), which can be generated by selection from combinatorial libraries for binders with high affinity and specificity to desired targets [8]. The use of affibody molecules for targeted delivery is advantageous in comparison with the traditional mAbs or antibody fragments in several ways. Their small size permits faster extravasation and better penetration into the tumors compared with mAbs, resulting in more efficient delivery of the cytotoxic payload to solid tumors. Clinical trials evaluating the HER2-targeting radiolabeled affibody molecules ABY-002 [9] and ABY-025 [10], did not find any toxicity or immunogenicity associated with this protein scaffold. The production of affibody molecules is possible in prokaryotic hosts with high yields, which ensures lower manufacturing costs. Genetic engineering allows for the molecular design of constructs with different valency, linker composition, and drug load. Conjugation of a drug to an affibody molecule can be performed site-specifically to a unique cysteine introduced into the affibody scaffold, providing affibody-drug conjugates (AffiDCs) with well-defined structural modifications.

The feasibility of targeted therapy using AffiDC containing a dimeric anti-HER2 affibody molecule Z_HER2:2891_, an albumin binding domain (ABD), and a maytansine derivative, mcDM1, has been demonstrated in several preclinical studies [11,12]. Maytansine is a plant-derived cytotoxic agent, which inhibits the assembly of microtubules by binding to tubulin at the rhizoxin binding site. Its derivative, DM1, is commonly used in antibody-drug conjugates for tumor-targeted delivery. In AffiDCs, DM1 is conjugated to the C-terminal cysteine residue via a non-cleavable maleimidocaproyl (mc) linker. The ABD was introduced into this construct to reduce renal excretion of the targeted drug by binding to serum albumin and increasing its bioavailability and tumor accumulation. The study by Andersen et al. proposed that the mechanism of half-life extension by ABD is based on indirect targeting of FcRn [13]. The ABD bound to human serum albumin (HSA) did not affect the binding of HSA to FcRn, which indicated that binding of ABD and FcRn to albumin were non-competitive and without allosteric effects interfering with the binding of one or the other molecules. The FcRn-binding site is located in domain III of HSA [14] and the ABD-binding site is located in domain II of HSA [15]. An in vivo dual-labelling experiment in rats showed that ^177^Lu-labelled ABD-(Z_HER2:342_)_2_ had a similar biodistribution profile and blood half-life as ^111^In-labelled rat serum albumin (RSA). No difference was observed in the elimination rate of ABD-(Z_HER2:342_)_2_ compared with RSA. Similarly, ABD-fused AffiDCs have a long half-time in blood, most likely due to FcRn salvation. The contribution of enhanced permeability and retention (EPR) effect for ABD-fused AffiDCs was studied by Altai et al. by comparing HER2-targeting (Z_HER2_)_2_-ABD-DM1 conjugate with the non-targeted (Z_taq_)_2_-ABD-DM1 control and by saturating the tumor uptake with an excess of (Z_HER2_)_2_-ABD-IAA [11]. In both cases, the accumulation in tumors was significantly (*p* < 0.01) lower compared with (Z_HER2_)_2_-ABD-DM1, which indicated the specificity of tumor targeting.

Treatment using this construct was effective and prolonged survival of mice bearing HER2-expressing SKOV3 xenografts [11]. The incorporation of mcDM1 increased the hydrophobicity of the construct resulting in an elevated liver uptake. This could be partially alleviated by introduction of a hydrophilic linker containing three glutamates between the ABD and mcDM1, which decrease liver uptake of the anti-HER2 AffiDCs [12]. This was an essential finding because it reduces the risk for hepatic toxicity, which is a common reason for the failure of drugs during clinical trials and sometimes also for withdrawal of approved drugs from the market [16]. Following studies demonstrated that the use of a triglutamate linker also reduced the hepatic uptake of a homologous construct containing a monomeric affibody molecule for HER2-targeting [17]. Furthermore, that study also demonstrated that the use of a monomeric instead of dimeric affibody molecule reduces the hepatic uptake further without affecting tumor targeting [17]. The optimal construct (designated Z_HER2:2891_-ABD-mcDM1) efficiently suppressed tumor growth and significantly prolonged survival of mice carrying SKOV3 xenografts [17]. To achieve an even higher efficient drug concentration in the tumors, Ding et al. designed and evaluated an AffiDC with three mcDM1 drugs separated by triglutamate linkers [18]. It was found that by increasing the DAR of an HER2-binding AffiDC from one mcDM1 to three, a 1.45-fold higher amount of mcDM1 could be delivered to the tumors. However, a significantly higher non-specific uptake in the liver, spleen, and bone was also observed. Overall, these studies have demonstrated that investigations of factors influencing the biodistribution of targeted, affibody-based drug conjugates, enable the finding of refined molecular design, which provides lower uptake in normal tissues, accompanied by a reduced systemic toxicity.

We hypothesized that further optimization of the biodistribution and targeting properties of AffiDCs might be achieved by modification of the inter-domain linker between the targeting affibody molecule and the ABD. Originally, this inter-domain linker consisted of four glycines and one serine (GGGGS or G_4_S). This is a variant of flexible (G_4_S)_n_-linkers commonly used in biotechnology [19]. The G_4_S linker was evaluated in the earlier studies with AffiDC and the constructs had a longer time in circulation compared with non-ABD-fused monomers [17,18]. We considered the flexibility of the linker as an essential property because it was expected that the construct should engage its receptor in vivo, predominantly in an albumin-bound state. In this case, the use of a flexible linker would help to avoid a steric hindrance due to the proximity of two bulky proteins. Flexible linkers should preferably contain small and hydrophilic amino acids [20], and both serine and glycine meet this requirement.

The linker length should also be considered when designing a multifunctional protein. A too short linker might result in insufficient domain separation and interference between adjacent domains. This could in turn cause incorrect folding of the domains [21,22,23] or impaired biological functions [24,25]. For example, the use of a single S_3_G linker in a homodimer of ADAPT6, another engineered scaffold protein, prevented correct folding of the constituents, while constructs including di- and trimeric linkers (S_3_G)_3_ and (S_3_G)_3_ provided more accurate folding of the domains, accompanied by an avidity-dependent increase in affinity [23]. On the other hand, too long linkers could possibly form secondary structure elements, which might affect biological function [26]. Besides length and flexibility, off-target interactions of linkers should be taken into account. Our earlier studies demonstrated that substitution of glycines for more hydrophilic serines in peptide-based mercaptoacetyl-containing chelators for ^99m^Tc resulted in the reduction in hepatobiliary excretion of monomeric affibody molecules [27]. Thus, it would be attractive to compare biodistribution of AffiDC with inter-domain linkers containing predominantly serines with the biodistribution of counterparts with linkers containing predominantly glycines.

In the current study, building blocks containing three serines and one glycine (SSSG or S_3_G) or three glycines and one serine (GGGS or G_3_S) were chosen for construction of the linker region. Considering that the linkers of natural proteins contain generally between 3 to 15 residues [20], variants having three (S_3_G) linker repeats Z_HER2:2891_-(S_3_G)_3_-ABD-mcDM1 or three (G_3_S) linker repeats Z_HER2:2891_-(G_3_S)_3_-ABD-mcDM1 were constructed and compared with the variant having a single (G_4_S) linker Z_HER2:2891_-G_4_S-ABD-mcDM1 that was tested before [17]. To enable quantitative assessment of cellular processing and biodistribution, the three AffiDC variants were extended with an N-terminal HEHEHE-tag for site-specific labeling with ^99m^Tc using tricarbonyl chemistry [28]. Binding specificity and affinity of the radiolabeled AffiDCs to HER2-expressing cancer cells and cellular processing after binding was evaluated in vitro. Biodistribution over time was evaluated in normal mice for the two new variants and was compared with the biodistribution of the previously tested variant, Z_HER2:2891_-G_4_S-ABD-mcDM1.

## 2. Materials and Methods

### 2.1. General

A BCA protein assay kit (Thermo Fisher Scientific, Waltham, MA, USA) was used to determine the concentrations of the proteins and drug conjugates. For measurement of radioactivity from samples, an automatic gamma-counter with a 3-inch NaI (TI) well detector (2480 Wizard, Wallac, Finland) was used. Unpaired 2-tailed *t*-test or ANOVA test with Bonferroni’s post-hoc analysis were used to determine significant differences (*p* < 0.05). Statistical analysis was carried out using Prism (version 9.0.0 for Windows; GraphPad Software, La Jolla, CA, USA).

### 2.2. Design, Expression, and Purification of the Affibody Constructs

Genes encoding Z_HER2:2891_-(G_3_S)_3_-ABD-Cys and Z_HER2:2891_-(S_3_G)_3_-ABD-Cys, were generated as follows: first, genes encoding Z_HER2:2891_ and ABD-Cys, with the desired linker composition were PCR amplified from Z_HER2:2891_-(G_4_S)-ABD-Cys [17]. A DNA sequence encoding Met-His-Glu-His-Glu-His-Glu (HEHEHE-tag) was added to the N-terminus of the gene encoding Z_HER2:2891_, allowing for radionuclide labeling. Second, the PCR-products were connected by overlap extension PCR. The final PCR-products were flanked by NdeI and HindIII restriction enzyme sites. The genes were inserted into the pET-26b(+) expression vector by restriction with NdeI and HindIII followed by ligation. The sequences of the expression cassettes were confirmed by DNA sequencing.

The affibody variants were produced in *Escherichia coli* BL21*(DE3) (New England Biolabs, Ipswich, MA, USA). The cells were expressed in Tryptic Soy Broth (30 g/L) with yeast extract (5 g/L) medium containing 50 mg/L kanamycin at 37 °C. Protein expression was induced by addition of 1 mM Isopropyl β-D-1-thiogalactopyranoside (Appolo Scientific, Stockport, UK) when OD_600_ was between 0.6 and 1. After 3 h incubation at 37 °C, the cells were harvested by centrifugation and lysed by sonication. The supernatants were collected by centrifugation at 17,000 rpm for 20 min at 4 °C and were filtered through a 0.45 µm Acrodisc syringe filter (Pall, Port Washington, NY, USA). Protein purification was performed as previously described by affinity chromatography on a HiTrap NHS Sepharose column (GE Healthcare, Uppsala, Sweden) with immobilized human serum albumin (HSA) [29] on an ÄKTA system (GE Healthcare Life Sciences, Uppsala, Sweden). Proteins eluted from the column were lyophilized.

### 2.3. Conjugation with mcDM1

The lyophilized proteins were reconstituted in phosphate-buffered saline (PBS, Ph = 6.5), followed by incubation with 5 mM Tris (2-carboxyethyl) phosphine (TCEP) for 1 h at 37 °C to reduce potentially oxidized cysteines. The proteins were passed over PD-10 size-exclusion columns (GE Healthcare) for removal of excess TCEP. The cytotoxic drug, mcDM1 (Levena Biopharma, San Diego, CA, USA), was dissolved in DMSO to a final concentration of 20 mM, and 0.5 molar equivalents of the affibody constructs were added, followed by incubation at room temperature overnight. On the following morning, buffer exchange was carried out on HPLC buffer A (0.1% Trifluoroacetic acid (TFA) in H_2_O). The conjugate was subsequently loaded on a Zorbax C18 SB column (Agilent, Santa Clara, CA, USA) and purification by reversed-phase chromatography was performed in high performance mode. After loading, the column was eluted with a 30 min linear gradient from 30% to 60% buffer B (0.1% TFA in Acetonitrile). The flow rate during purification was 3 mL/min. Eluted fractions containing conjugates were pooled. This was followed by lyophilization, after which the samples were reconstituted in PBS (pH = 7.4) and were frozen to −20 °C until used.

Non-toxic control constructs were also generated by alkylation of the C-terminal cysteines of Z_HER2:2891_-(G_3_S)_3_-ABD-Cys and Z_HER2:2891_-(S_3_G)_3_-ABD-Cys with 2-Iodoacetamide (IAA). Purification of the non-toxic controls was carried out as previously described [18]. After purification, the controls were lyophilized, followed by reconstitution in PBS, (pH = 7.4) and frozen to −20 °C until used. Purified conjugates were analyzed by sodium dodecyl sulfate–polyacrylamide gel electrophoresis (SDS-PAGE), where separation took place under reducing conditions on a NuPAGE Bis-Tris gel (4–12%). The conjugates were also subjected to analytical size-exclusion chromatography. The column used was a Superdex 75, 5/150 column (GE Healthcare). PBS was used as a running buffer and the flow rate was 0.45 mL/min. The purified conjugates were analyzed by ESI-TOF mass spectrometry (Agilent). The AffiDCs were analyzed by RP-HPLC (Zorbax C18 SB, Agilent) using a 30 min gradient from 30% to 60% HPLC buffer B (0.1% TFA in Acetonitrile) at a flow rate of 1 mL/min. The purity was determined by calculating the area-under curve from all peaks in the recorded chromatograms.

### 2.4. Analysis of Affinity to HER2, HSA, and MSA

A Biacore T200 instrument (Biacore Life Science, GE Healthcare, Uppsala, Sweden) was used for biosensor analysis. The recombinantly produced extracellular domain of human HER2 (Sino Biological, Beijing, China) was immobilized in one flow channel on a CM5 sensor chip by amine coupling in sodium acetate buffer (pH = 4.5). Similarly, HSA (Novozymes, Bagsvaerd, Denmark) and MSA (Sigma-Aldrich, St. Louis, MO, USA) was immobilized on different flow channels on the same CM5 chip. A control was created by activation and deactivation of a fourth flow channel on the same chip. PBS supplemented with 0.05% Tween 20 (PBST, pH = 7.4) was used as running buffer (50 μL/min). The regeneration of the chip was carried out by injecting 20 mM HCl for 30 s. The kinetics of binding was determined from the recorded sensorgrams by the Biacore T200 Evaluation Software using a 1:1 interaction model.

### 2.5. Cell Culture

SKOV3, BT474, SKBR3, AU565, MCF7, and A549 cell lines were obtained from American Type Culture Collection (ATCC) and cultured in McCoy’s 5A medium (SKOV3, SKBR3), RPMI-1640 medium (AU565, BT474), or Dulbecco’s modified Eagle medium (MCF7, A549) supplemented with 10% FBS (20% for BT474 cells) (Sigma-Aldrich), 2 mM L-glutamine, 100 IU/mL penicillin, and 100 µg/mL streptomycin in a humidified incubator at 37 °C in 5% CO_2_ atmosphere.

### 2.6. In Vitro Cytotoxicity Analysis

All cell lines were counted and seeded in 96-well plates (5000 cells/well for SKBR3, AU565, MCF7 and A549; 2000 cells/well for SKOV3) and were allowed to attach for 24 h. Subsequently, the medium was changed to fresh medium containing serial dilutions of the affibody constructs and were incubated for another 72 h. Cell viability was evaluated by a cell counting kit-8 (CCK-8, Sigma-Aldrich). The obtained values for viability were plotted and analyzed using Prism (GraphPad Software, La Jolla, CA, USA).

### 2.7. Radiolabeling and In Vitro Stability

The CRS kits for production of tricarbonyl technetium were purchased from the Center for Radiopharmaceutical Sciences (PSI, Villigen, Switzerland). Purification was performed using NAP-5 size-exclusion columns (GE Healthcare, UK). Instant thin-layer chromatography (iTLC) analysis was performed using iTLC silica gel strips (Varian, Lake Forest, CA, USA). Analysis of the iTLC strips were performed using a Cyclone Storage Phosphor System (PerkinElmer, Waltham, MA, USA) and OptiQuant image analysis software (PerkinElmer, Waltham, MA, USA). Site-specific radiolabeling of the affibody-mcDM1 conjugates with [^99m^Tc][Tc(CO)_3_(H_2_O)_3_]^+^ (tricarbonyl technetium) on the N-terminal HEHEHE amino acid sequence was performed as described earlier [17]. In brief, technetium-99m pertechnetate [^99m^Tc]TcO_4_^−^ was obtained from a ^99^Mo/^99m^Tc generator (Mallinckrodt, Petten, The Netherlands). To produce the [^99m^Tc(CO)_3_(H_2_O)_3_]^+^ precursor, 500 μL (3.2–4.7 GBq) of [^99m^Tc]TcO_4_^−^ was added to a CRS kit vial and incubated at 100 °C for 30 min. To neutralize [^99m^Tc][Tc(CO)_3_(H_2_O)_3_]^+^, the same volume of 0.1 M HCl was added after the incubation. The solution of neutralized [^99m^Tc][Tc(CO)_3_(H_2_O)_3_]^+^ (19–26 µL, 80 MBq) was mixed with 40 µg (2.7–2.8 nmol, 29–37 μL in PBS) of an affibody-mcDM1 conjugate and incubated at 60 °C for 60 min. Then, a 1000-fold molar excess of histidine (2.7–2.8 μmol, 42–43 µL of 10 mg/mL in PBS) was added and incubated at 60 °C for 10 min to remove loosely bound tricarbonyl technetium. The radiolabeled affibody-mcDM1 conjugates were purified by passing through a NAP-5 size-exclusion column pre-equilibrated with 1% bovine serum albumin (BSA) in PBS and eluted with PBS.

To evaluate the stability of labeling, the radiolabeled affibody-mcDM1 conjugates were incubated with a 1000-fold molar excess of histidine in PBS or PBS only (control) at room temperature for up to 4 h.

The radiochemical yield and purity were measured using radio-iTLC eluted with PBS. In this system, the radiolabeled affibody-mcDM1 conjugates stay at the application point, all forms of free radionuclides move with the solvent front.

### 2.8. In Vitro Binding Specificity and Cellular Processing

The in vitro specificity of ^99m^Tc-labeled affibody-mcDM1 conjugates binding to HER2 was tested using SKOV3, BT474, and SKBR3 cell lines as described earlier [30]. In brief, cells were seeded in 6-well plates at a density of 1 × 10^6^ cells per well one day before the experiment. Non-radiolabeled affibody-mcDM1 conjugate (1000 nM in 500 μL) was added to one set of wells (n = 3) to saturate HER2 receptors on the cells, and an equal volume of culture medium only was added to another set of wells (n = 3). After incubation at room temperature for 30 min, a solution of the same ^99m^Tc-labeled affibody-mcDM1 conjugate (4 nM in 500 μL) was added to both sets of wells, followed by incubation at 37 °C for 60 min. The medium was collected, followed by washing of the cells with PBS (1 mL). The cells were lysed by incubation with 1 M NaOH (1 mL) at 37 °C for 15–30 min and detached with a rubber scraper. The cell lysates were collected, followed by washing with 1 mL of 1 M NaOH and collected. The activity of the medium and the cell fractions was measured using a gamma spectrometer and calculated for cell-associated activity.

To evaluate the cellular processing of ^99m^Tc-labeled affibody-mcDM1 conjugates after binding to HER2-expressing cells, an experiment was performed as described previously [30]. In brief, SKOV3 and BT474 cells were seeded in 35 mm dishes at a density of 8 × 10^5^ cells/dish one day before the experiment. The medium was aspirated, followed by addition of ^99m^Tc-labeled affibody-mcDM1 conjugates in culture medium (2 nM). The cells were incubated at 37 °C in 5% CO_2_ atmosphere. At determined time points (1, 2, 4, 6 and 24 h), a set of dishes (n = 3) was analyzed. First, the medium was collected followed by washing with ice-cold PBS (1 mL). Second, 0.2 M glycine buffer containing 4 M urea (pH 2.0) (1 mL) was added to cells for 5 min on ice to collect activity bound to cell membrane. The solution was collected and added to a wash using the same buffer (1 mL). Third, 1 M NaOH (1 mL) was added to the cells for 30 min at 37 °C. Cell debris containing internalized activity were collected by a rubber scraper and the dishes were washed with the same buffer (1 mL) and collected. The activity of the collected medium, the membrane-bound activity and the internalized activity was measured using an automatic gamma spectrometer.

### 2.9. Affinity Measurement to Cells

To measure the binding affinity of ^99m^Tc-labeled affibody-mcDM1 conjugates to SKOV3 cells with high HER2 expression, a Ligandtracer Yellow instrument (Ridgeview Instruments, Vänge, Sweden) was used as described earlier [17]. In brief, one day before the experiment, 2 × 10^6^ cells were seeded to one side of an 89-mm petri dish and were incubated at 37 °C overnight. The measurements were performed at room temperature. Several concentrations (1, 2 and 5 nM) of the ^99m^Tc-labeled affibody-mcDM1 conjugates were added stepwise to measure the association phase. To measure the dissociation phase, the medium containing ^99m^Tc-labeled affibody-mcDM1 conjugates was exchanged to complete medium lacking conjugate. The data were collected, corrected for nuclide decay, and analyzed using Tracedrawer software (Ridgeview Instruments) for the equilibrium dissociation constants (K_D_). Interaction map analysis (Ridgeview Diagnostics, Uppsala, Sweden) was performed to estimate the interaction heterogeneity.

### 2.10. Biodistribution in NMRI Mice

To evaluate the biodistribution of ^99m^Tc-labeled affibody-mcDM1 conjugates, 36 female NMRI mice were randomized to 9 groups including four animals each. The average animal weight was 28.3 ± 2.6 g. The mice were intravenously (i.v.) injected with 6 μg (60 kBq for 4 h, 640 kBq for 24 h, and 10.2 MBq for 48 h time points) of ^99m^Tc-labeled affibody-mcDM1 conjugates, and the biodistribution was measured 4, 24, and 48 h post injection (p.i.). At each time point, one group of mice injected with ^99m^Tc-Z_HER2:2891_-(G_3_S)_3_-ABD-mcDM1, ^99m^Tc-Z_HER2:2891_-(S_3_G)_3_-ABD-mcDM1 or ^99m^Tc-Z_HER2:2891_-G_4_S-ABD-mcDM1 was weighed and received an intraperitoneal (i.p.) injection of a ketamine–xylazine anesthesia (30 μL/gram body weight; ketamine 10 mg/mL; xylazine 1 mg/mL). Anaesthetized mice were sacrificed by cardiac puncture. The blood, organs, and tissues were collected and weighed and the activity was measured using an automated gamma-counter. The activity uptake was calculated as the percentage of injected dose per gram of sample (%ID/g).

## 3. Results

### 3.1. Production and Biochemical Characterization of the AffiDC

**Figure 1:**
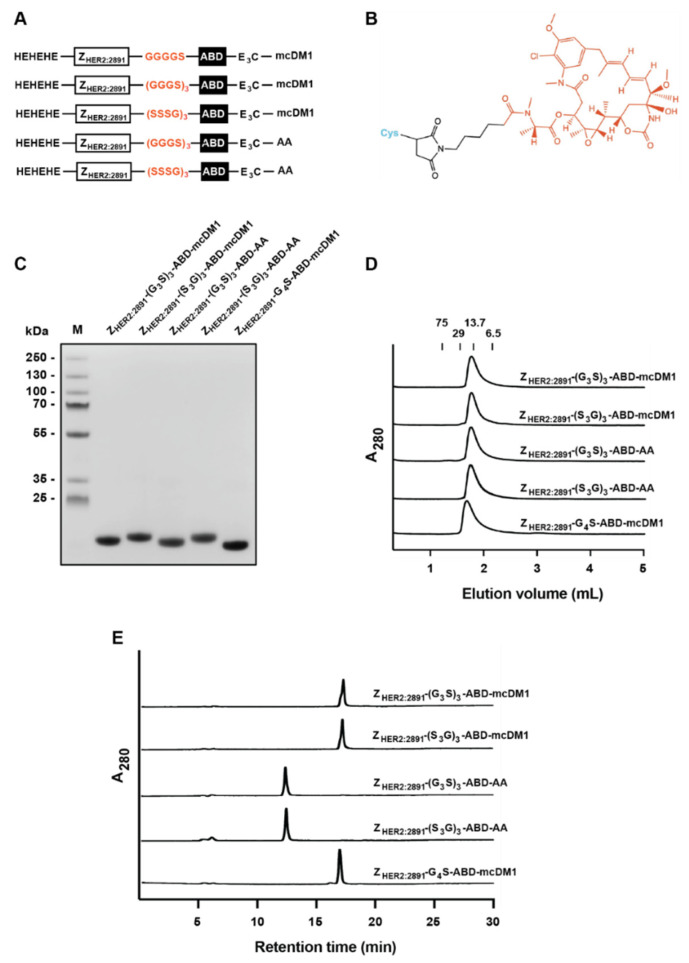
Schematic representation and initial characterization. (**A**) A schematic representation of the constructs. All constructs contained the Z_HER2:2891_ affibody molecule and ABD connected by different linkers highlighted in yellow. (**B**) The structure of mcDM1 with DM1 (red), the maleimidocaproyl linker (black) and the protein’s C-terminal cysteine (blue). (**C**) The gel obtained after sodium dodecyl sulfate–polyacrylamide gel electrophoresis. The names of the constructs are indicated above each lane. In lane M, a set of marker proteins were separated, and the molecular weight of those proteins are indicated to the left. (**D**) The panel shows the chromatograms obtained during analytical size-exclusion chromatography. The numbers above the chromatograms indicate the elution volumes of protein standards, with the molecular weights indicated in kDa. (**E**) The panel shows the chromatograms obtained during RP-HPLC analysis.

The affibody constructs with different linkers ((G_3_S)_3_, (S_3_G)_3_, or G_4_S) are shown in Figure 1A. All constructs were recombinantly expressed in *Escherichia coli* and were purified by affinity chromatography using human serum albumin (HSA) as ligand. The constructs contained a unique cysteine in the C-terminal end, which was used for conjugation of the cytotoxic drug DM1 via a non-cleavable maleimidocaproyl (mc)-linker (Figure 1B). Non-toxic control constructs were also generated by alkylation of the C-terminal cysteine in Z_HER2:2891_-(G_3_S)_3_-ABD and Z_HER2:2891_-(S_3_G)_3_-ABD. After DM1 conjugation or alkylation, the constructs were purified by RP-HPLC (reversed-phase high-performance liquid chromatography) to remove unconjugated affibody and DM1. The final products were subjected to SDS-PAGE. The resulting gel showed constructs with the expected molecular weights (Figure 1C). By visual inspection of the gel in Figure 1C it could be concluded that the conjugates were of high purity.

Analysis by size-exclusion chromatography showed that all five constructs were eluted as single peaks, showing that they were in a monomeric state and that degradation products were absent (Figure 1D). The purity of all conjugates was above 95%, determined by analytical RP-HPLC (Figure 1E). The non-toxic controls, Z_HER2:2891_-(G_3_S)_3_-ABD-AA, and Z_HER2:2891_-(S_3_G)_3_-ABD-AA were eluted earlier than the conjugates with mcDM1 in analytical RP-HPLC, suggesting an increase in hydrophobicity after conjugation of DM1. Moreover, the molecular weight of the conjugates was determined by mass spectrometry and showed constructs matching the theoretical molecular weight (Figure S1).

### 3.2. Evaluation of Binding Affinity to HER2, HSA, and MSA

**Figure 2:**
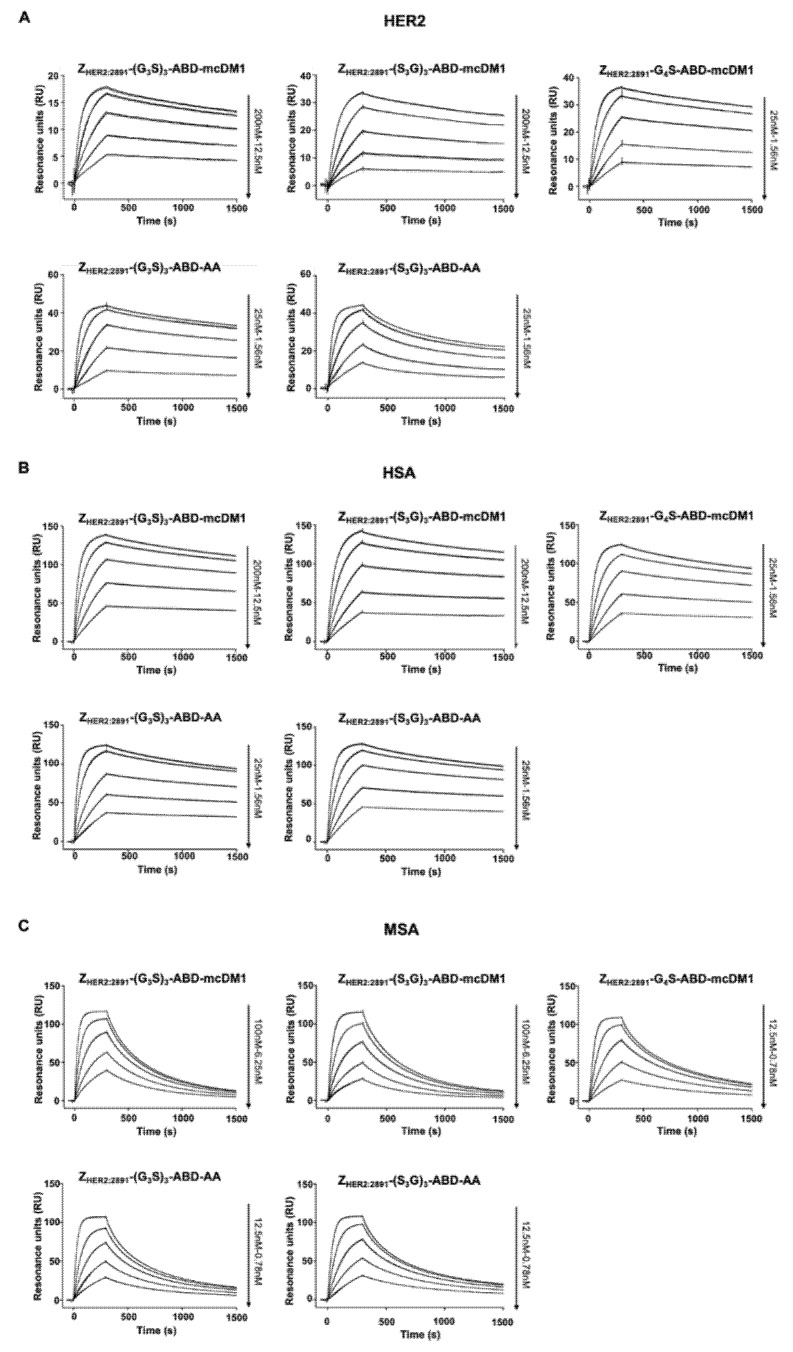
Biosensor analysis. Dilution series of the conjugates were injected over flow cells with immobilized HER2 (**A**), HSA (**B**), or MSA (**C**). All concentrations were injected twice and each panel is an overlay of the sensorgrams for each combination of ligand and analyte. The number to the right indicates the concentrations of the dilution series.

In order to investigate if affibody linker composition affected the ability of Z_HER2:2891_ to interact with HER2, all constructs were injected in a biosensor over a chip surface with immobilized HER2 (Figure 2A). The dissociation rates (k_d_) of the three conjugates, Z_HER2:2891_-(G_3_S)_3_-ABD-mcDM1, Z_HER2:2891_-(S_3_G)_3_-ABD-mcDM1, and Z_HER2:2891_-G_4_S-ABD-mcDM1, were found to be similar and ranged from 1.9 to 2.5 × 10^−4^ s^−1^. The association rates (k_a_) for Z_HER2:2891_-(G_3_S)_3_-ABD-mcDM1 and Z_HER2:2891_-(S_3_G)_3_-ABD-mcDM1 were slower (9.7 × 10^4^ 1/Ms and 7.1 × 10^4^ 1/Ms, respectively) in comparison with Z_HER2:2891_-G_4_S-ABD-mcDM1 (7.5 × 10^5^ 1/Ms) and the non-toxic controls Z_HER2:2891_-(G_3_S)_3_-ABD-AA (1.2 × 10^6^ 1/Ms) and Z_HER2:2891_-(S_3_G)_3_-ABD-AA (8.8 × 10^5^ 1/Ms). The equilibrium dissociation constants (K_D_) were calculated and are displayed in Table 1.

To further characterize the conjugates, their binding to HSA and mouse serum albumin (MSA) was analyzed. Dilution series of the constructs were injected over surfaces with immobilized serum albumins (Figure 2B,C). From the recorded sensorgrams, the kinetic parameters were determined followed by calculation of the equilibrium dissociation constants (K_D_ values) (Table 1). For Z_HER2:2891_-(G_3_S)_3_-ABD-mcDM1 and Z_HER2:2891_-(S_3_G)_3_-ABD-mcDM1, the affinity was weaker to both HSA and MSA compared with Z_HER2:2891_-G_4_S-ABD-mcDM1 and the non-toxic control constructs due to slower on-rates.

### 3.3. In Vitro Cytotoxicity Analysis

**Figure 3:**
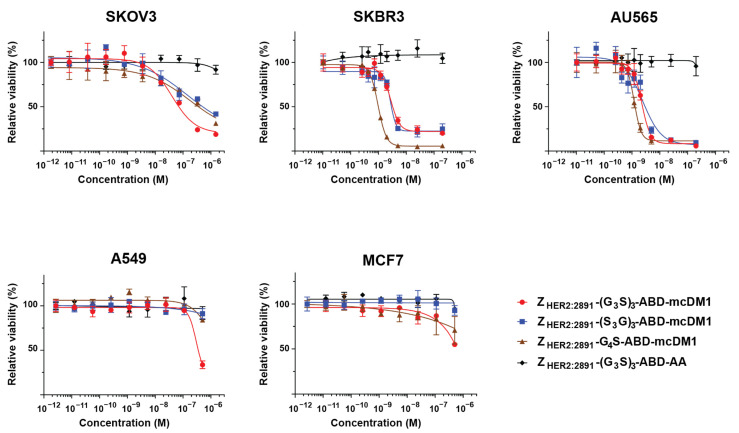
Analysis of the cytotoxicity of the affibody-mcDM1 conjugates. The relative viability of the different cell lines was plotted on the *Y*-axis as a function of the protein concentration on the *X*-axis. The viability at the lowest concentration for each dilution series was set to 100%. Each data point was performed in quadruplicate experiments, and the mean value was plotted with error bars corresponding to 1 SD.

To investigate the correlation between cytotoxic effect and HER2 expression level, different cell lines with a low, medium, or high level of HER2 expression were cultured with dilution series of the conjugates, followed by calculation of cell viability (Figure 3, Table 2). For high HER2-expressing cells (SKOV3, SKBR3, and AU565), the constructs Z_HER2:2891_-(G_3_S)_3_-ABD-mcDM1, Z_HER2:2891_-(S_3_G)_3_-ABD-mcDM1, and Z_HER2:2891_-G_4_S-ABD-mcDM1 showed a dose-dependent cytotoxic effect. The IC_50_ values were similar for the AU565 cell line, ranging from 1.2 to 2.4 nM. For the SKBR3 cell line, the cytotoxic effect was slightly weaker for the constructs with the (G_3_S)_3_ and the (S_3_G) linkers compared with the effect of the construct with the G_4_S linker. For the SKOV3 cell line, the curves had a different appearance, with a flatter shape. The IC_50_ value was 48 nM for Z_HER2:2891_-(G_3_S)_3_-ABD-mcDM1, while the IC_50_ for Z_HER2:2891_-(S_3_G)_3_-ABD-mcDM1 and Z_HER2:2891_-G_4_S-ABD-mcDM1 were found to be 150 nM and 180 nM, respectively. Both A549 and MCF7 expressed a low/medium level of HER2. They were unaffected by all constructs except for the highest concentration. The non-toxic control Z_HER2:2891_-(G_3_S)_3_-ABD-AA did not affect viability of any of the cell lines.

### 3.4. Radiolabeling

The affibody-mcDM1 conjugates were labeled with [^99m^Tc][Tc(CO)_3_(H_2_O)_3_]^+^ to evaluate the binding properties to HER2-expressing cells in vitro and biodistribution. This approach provides a residualizing label. After the radiolabeling reaction, the conjugates were incubated with a 1000-fold molar excess of histidine at 60 °C for 10 min, with minor release of activity (between 1–4%) observed for the three conjugates. The labeling resulted in over 93% radiochemical yield for all three conjugates and the radiochemical purity after size-exclusion chromatography purification was over 98% (n = 2). No significant release of activity from the radiolabeled conjugates was observed after 4 h incubation with a large molar excess of histidine (Table 3).

### 3.5. In Vitro Specificity, Cellular Processing, and Affinity Measurement

**Figure 4:**
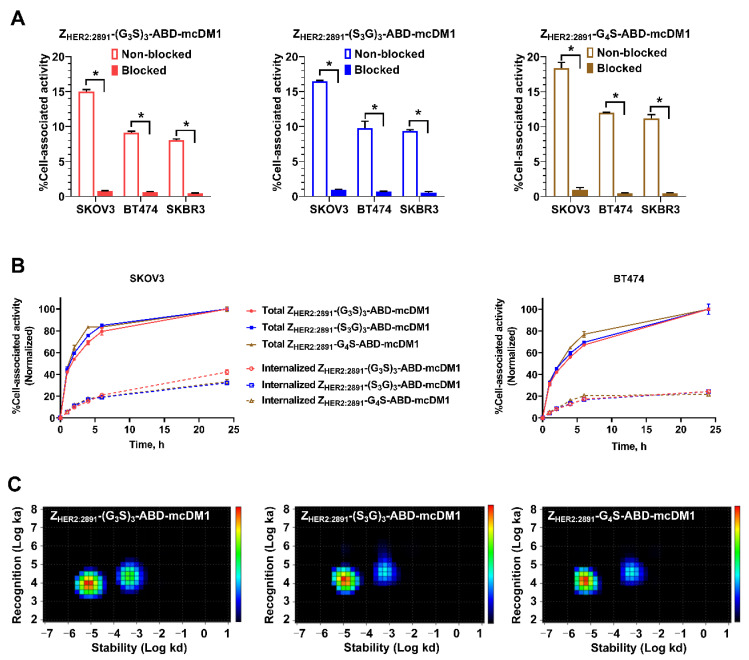
Cell binding specificity, cellular processing, and interaction map of ^99m^Tc-labeled affibody-mcDM1 conjugates. (**A**) SKOV3, BT474 and SKBR3 cells were incubated with ^99m^Tc-Z_HER2:2891_-(G_3_S)_3_-ABD-mcDM1, ^99m^Tc-Z_HER2:2891_-(S_3_G)_3_-ABD-mcDM1, or ^99m^Tc-Z_HER2:2891_-G_4_S-ABD-mcDM1 in the presence or absence of a 500-fold molar excess of the same non-labeled conjugate, blocked or non-blocked, respectively. The total cell-bound activity is shown on the *Y*-axis as a percentage of the total added activity. Asterisk (*) correspond to significant differences (*p* < 0.05). (**B**) Processing and internalization of the conjugates (2 nM) during continuous incubation with SKOV3 and BT474 cells for 24 h. Data were normalized to the value at 24 h taken as 100% and are presented as the average from three samples ± SD. (**C**) Interaction between ^99m^Tc-labeled affibody-mcDM1 conjugates and SKOV3 cells is shown as a heat map. Data are representative from duplicate experiments.

To investigate the binding specificity to HER2-expressing cells, SKOV3, BT474, and SKBR3 cells were incubated with ^99m^Tc-labeled affibody-mcDM1 conjugates. When HER2 receptors were pre-saturated with the non-labeled affibody-mcDM1 conjugates, the uptake of activity was significantly decreased (*p* < 0.05) (Figure 4A). These results indicate specific HER2-mediated binding of all ^99m^Tc-labeled affibody-mcDM1 conjugates to SKOV3, BT474, and SKBR3 cells.

To evaluate the rate of processing and internalization, SKOV3 and BT474 cells were incubated with ^99m^Tc-labeled affibody-mcDM1 conjugates for 24 h and the membrane-bound and internalized activity was measured as a function of time (Figure 4B). Overall, a similar pattern with a minor impact of linker on internalization was observed. The cell-associated activity and internalized activity increased over time for three conjugates in both SKOV3 and BT474 cells. After incubation for 24 h, the internalized activity for the conjugates with (G_3_S)_3_, (S_3_G)_3,_ and G_4_S linkers reached 42.2 ± 2.1%, 32.3 ± 1.6%, and 33.3 ± 2.1% of the total cell-associated activity in SKOV3 cells and 24.3 ± 0.4%, 24.2 ± 1.3%, and 21.7 ± 0.6% in BT474 cells, respectively.

The binding kinetics of the ^99m^Tc-labeled affibody-mcDM1 conjugates to SKOV3 cells were investigated in real-time using a LigandTracer Yellow instrument. For all three conjugates, two peaks were identified, indicating two interactions, one with higher affinity and one with lower affinity (Figure 4C). The corresponding equilibrium dissociation constants (K*_D_*) values are shown in Table 4. The affinities for the conjugates with (G_3_S)_3_ and (S_3_G)_3_ linkers were slightly lower than for the conjugate with a G_4_S linker.

### 3.6. Biodistribution

To determine the biodistribution of affibody-mcDM1 conjugates over time, NMRI mice were injected with ^99m^Tc-Z_HER2:2891_-(G_3_S)_3_-ABD-mcDM1, ^99m^Tc-Z_HER2:2891_-(S_3_G)_3_-ABD-mcDM1, or ^99m^Tc-Z_HER2:2891_-G_4_S-ABD-mcDM1, and activity uptake in organs and tissues was measured at 4, 24, and 48 h post injection (p.i.) (Table 5). 

For all three time points, the majority of the activity was in kidneys, which suggested excretion via the urinary system.

**Figure 5:**
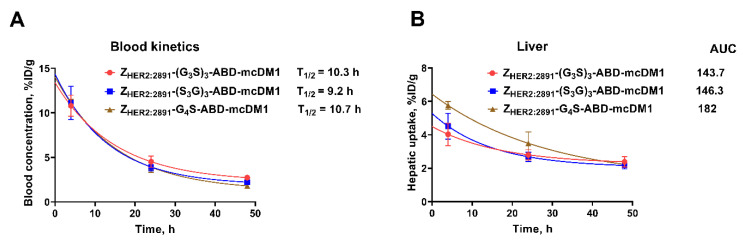
Blood kinetics (**A**) and liver uptake of activity (**B**) plotted as a function of time.

A prolonged blood retention was observed for all three conjugates. At 4 and 24 h, no differences in blood activity was observed between the conjugates. By 48 h, the conjugates with the (G_3_S)_3_ and (S_3_G)_3_ linkers had significantly (*p* < 0.05) higher activity in blood than the conjugate with the G_4_S linker. The activity in blood was plotted as a function of time to calculate the plasma half-life (T_1/2_) (Figure 5A). The conjugate with the (S_3_G)_3_ linker had slightly shorter blood half-life (T_1/2_ = 9.2 h) than the conjugates with (G_3_S)_3_ (T_1/2_ = 10.3 h) and with G_4_S (T_1/2_ = 10.7 h) linkers, with overlapping confidence intervals.

By 4 h, the conjugate with the (G_3_S)_3_ linker had the lowest uptake in salivary gland, lung, liver, spleen, stomach, muscle, and bone among the three conjugates. The conjugates with (G_3_S)_3_ and (S_3_G)_3_ linkers had significantly (*p* < 0.05) reduced liver and stomach uptake compared with the conjugate with the G_4_S linker.

By 24 h, the uptake in all organs was similar (*p* > 0.05) for the three conjugates.

By 48 h, there was a minor but significant difference between the uptake of three conjugates in several organs, including salivary gland, lung, spleen, stomach, and muscle.

The activity in liver was plotted as a function of time and calculated for area under curve (AUC) (Figure 5B), which was noticeably smaller for the conjugates with the (G_3_S)_3_ linker (1.27-fold) and the (S_3_G)_3_ linker (1.25-fold) than for the conjugate with the G_4_S linker.

## 4. Discussion

Targeted therapeutics carrying cytotoxic drugs allow for selective delivery of a payload to tumors and reduction in toxicity to normal organs and tissues when compared with standard chemotherapy approaches. Antibody-drug conjugates are a well-established type of targeted anti-cancer therapeutics, and several ADCs has received approval for clinical use during the last decade [31,32]. This has been made possible by nearly 50 years of active research to understand factors that influence ADC efficacy, including selection of drugs, linkers for their coupling and conjugation methods, and by careful optimization of the molecular design of the conjugates [33,34].

Targeted drug conjugates based on affibody molecules have appeared only recently. Affibody molecules are easy to engineer, which enables facile development of multivalent and multimeric constructs with optimized biodistribution and targeting characteristics [8]. However, extensive information concerning the structure-function relationship of affibody-based drug conjugates is still required to utilize their full potential as anti-cancer drugs.

An attractive approach to study the structure–function relationship of proteins and protein-based drug conjugates is the use of radioactive labeling, which permits the acquisition of quantitative information, such as the rate of internalization and assessment of biodistribution in blood and other organs and tissues. Selection of a suitable label is essential for such studies. We applied a site-specific labeling of the HEHEHE-tag of the constructs using [^99m^Tc][Tc(CO)_3_(H_2_O)_3_]^+^ [28]. This labeling method should have minimal impact on the binding properties of affibody molecules, since the label and the binding site of the affibody molecule are spatially separated. This label is residualizing, i.e., it is trapped in the lysosomal compartment after internalization and proteolytic degradation, which enables the evaluation of internalization rates of the constructs. Furthermore, biodistribution of affibody molecules labeled using this approach and other site-specific labeling methods is similar [28,35], which suggest that this method does not modify in vivo properties of the affibody molecules. Application of this radiolabeling method has earlier enabled elucidation of the impact of valency [17], the linker between the ABD and the drug [12], and the drug load [18] on the targeting properties of AffiDC. Collectively, the papers by Xu et al. and Ding et al. showed that it was possible to substantially reduce liver uptake of the conjugates to minimize the risk of hepatotoxicity. The current study aimed to further elucidate the structure/function relationship of AffiDCs by investigation of the influence of inter-domain linkers on the biodistribution.

The measurements of affinity to HER2 using SPR (Figure 2 and Table 1) and binding to living HER2-expressing cells using LigandTracer methodology (Figure 4C, Table 4) revealed a similar pattern. The affinity of Z_HER2:2891_-G_4_S-ABD-mcDM1 to HER2 was higher than the affinity of the drug-conjugated constructs with longer linkers, (S_3_G)_3_ and (G_3_S)_3_. Furthermore, the affinity of the conjugates with the (S_3_G)_3_ and (G_3_S)_3_ linkers to both murine and human serum albumins was also reduced compared with the conjugate with the G_4_S linker (Table 1). This might indicate that the increase in the length of flexible linkers causes inter-domain interaction and leads to a change in the constructs’ conformations, which creates steric hindrance for binding to HER2. Interestingly, the loss of affinity was not observed when the C-terminal cysteines were alkylated instead of conjugated with mcDM1. It suggests that the presence of mcDM1 in the constructs with longer linkers interferes with the proper folding by e.g., hydrophobic interactions with amino acids in the affibody molecule. It has to be noted that the affinity to HER2 of both Z_HER2:2891_-(G_3_S)_3_-ABD-mcDM1 and Z_HER2:2891_-(S_3_G)_3_-ABD-mcDM1 remains strong, in the low nanomolar range, which should be sufficient for efficient targeting of HER2-positive tumors in therapeutic applications.

Binding to albumin is also essential to prevent rapid renal excretion of the targeting constructs to increase their bioavailability. In principle, a reduction in affinity to albumin might result in a more rapid elimination of the targeting constructs from circulation. However, since the albumin concentration in blood is 0.6 mM, it can be expected that the constructs are predominantly present as bound to albumin in vivo. Indeed, there was no significant difference between the plasma half-lives of the variants in mice, even though their affinity to murine serum albumin ranged from 7.4 × 10^−10^ to 1.1 × 10^−8^ M (Figure 5A). We have previously compared the biodistribution of two constructs, (Z_HER2:2891_)_2_-ABD-DM1 and (Z_HER2:2891_)_2_-DM1, and found that at 4 h post injection the level of activity in tumors was very similar [11], indicating that the binding of albumin to affibody-ABD-DM1 did not interfere with accumulation in tumors and provided a higher amount of drug delivered to tumors due to longer half-life in blood (Figure 4B). We have previously evaluated several ABD-fused affibody-drug conjugates in therapeutic studies [11,17], where they have shown to efficiently suppress tumor growth in comparison with the control and cause complete tumor regression in some mice, indicating that fusion of affibody molecules to ABD is a promising strategy for tumor-targeted drug delivery.

Efficient internalization of the DM1-conjugates is an essential property because it creates the precondition for intracellular release of the drug and the blocking of tubulin polymerization. The data in Figure 4 indicate that binding and subsequent internalization of ABD-fused AffiDCs were predominantly HER2 mediated. ABD-fused AffiDCs bound to HER2 on living cells in a HER2-specific manner (Figure 4A). The internalization experiment (Figure 4B) allows for discrimination of the internalized and membrane-bound fractions [30] and is performed using a residualizing tricarbonyl technetium-99m label, meaning that the radiocatabolites do not diffuse through the cell membranes and are retained inside the cells after HER2-mediated endocytosis and protein degradation in lysosomes. The internalization experiment (Figure 4B) showed that ca. 30–40% of total cell bound-activity was internalized by 24 h, which is very similar to the internalization level of ^99m^Tc(CO)_3_-labeled affibody molecules (ca. 30%) [28]. The results of the cytotoxicity experiment (Figure 3) show that there are differences between cytotoxicity of DM1-conjugated constructs in comparison with the control construct without DM1. Our experiments demonstrate that the internalization is cell line-dependent, where the rate was higher for the ovarian cancer SKOV3 cell line compared with the breast cancer BT474 cells line (Figure 4B). Interestingly, the linker composition had no effect on the rate of internalization by BT474 cells, but the rate of internalization of ^99m^Tc-Z_HER2:2891_-(G_3_S)_3_-ABD-mcDM1 by SKOV3 cells was significantly faster. This correlated with a higher toxicity (lower IC_50_ value) of this conjugate to SKOV3 cells (Table 2).

For all constructs, the cytotoxic effect (Figure 3) was clearly dependent on the HER2 expression level of the cancer cell lines. In the case of high expression (SKOV3, SKBR3 and AU565), the cytotoxicity was dose dependent with half-inhibiting concentrations in the nanomolar range (Table 2). The cytotoxic effect on cell lines with low/medium HER2 expression (A549 and MCF7) was much less pronounced (Figure 3). Taken together, these observations confirm the targeted character of the cytotoxic effect. In the case of the SKBR3 and the AU565 cell lines, Z_HER2:2891_-G_4_S-ABD-mcDM1 demonstrated the highest cytotoxicity, which correlated with its higher affinity to HER2. However, the cytotoxicity to the SKOV3 cell line was different, where Z_HER2:2891_-(G_3_S)_3_-ABD-mcDM1 demonstrated a stronger effect compared with Z_HER2:2891_-G_4_S-ABD-mcDM1. It has to be noted that the SKOV3 cell line demonstrated strong resistance to anti-HER2 targeting drugs and toxins based on affibody molecules and other scaffold proteins, such as ADAPTs [12,36,37] and can be considered as the most challenging case for such treatment among the cell lines investigated in this study. The finding, in this study, of a molecular design, which increases the cytotoxic effect against this cell line, is an important achievement.

The goal of any targeted therapy is an enhancement of the anti-tumor effect combined with a reduction in systemic toxicity. The latter can be achieved by molecular design to minimize the uptake in normal organs and tissues. In this study, the direct side-by-side comparison of constructs with different intra-domain linkers demonstrates that the use of (S_3_G)_3_ and (G_3_S)_3_ linkers resulted in a significant decrease in the liver uptake compared with the use of a G_4_S linker (Table 5). This is an important achievement, since the hepatic toxicity and associated drug-induced liver injury is a side effect that may result in failure during clinical trials and sometimes even in withdrawal of drugs already approved for clinical use [16]. Earlier studies with affibody molecules and affibody-drug conjugates have demonstrated that the hepatic uptake correlates with the presence of “lipophilic” patches on water-exposed surfaces of these proteins and might be counteracted by incorporation of hydrophilic amino acids [12,38]. It is reasonable to suppose that an increased content of serines in (S_3_G)_3_ and (G_3_S)_3_ linkers causes the lower hepatic uptake of the new constructs. Overall, the lessons from this study suggest that the inter-domain linker between the targeting affibody molecule and ABD should not be exceedingly long to provide higher affinity and should contain additional serines to suppress hepatic uptake.

## 5. Conclusions

The length and composition of the inter-domain linkers have a clear effect on the targeting and biodistribution properties of mertansin-conjugated, and ABD-fused, affibody molecules. This study suggests that a 12 amino acid linker is associated with lower affinity to HER2 and weaker cytotoxic effect in vitro, compared with a 5 amino acid linker. Most likely, this phenomenon is caused by unfavorable conformation changes, which are enhanced by the presence of DM1 on the C-terminus of the ABD. At the same time, the longer linker with a high serine content provides lower liver uptake, potentially reducing hepatic toxicity.

## Figures and Tables

**Table 1:** Affinity constants for Affibody-mcDM1 conjugates.

Analytes	Ligand	k_a_ (M^−1^·s^−1^)	k_d_ (s^−1^)	K_D_ (M)
Z_HER2:2891_-(G_3_S)_3_-ABD-mcDM1	HER2	9.7 × 10^4^	2.2 × 10^−4^	2.3 × 10^−9^
	HSA	1.1 × 10^5^	1.6 × 10^−4^	1.4 × 10^−9^
	MSA	4.5 × 10^5^	3.1 × 10^−3^	6.8 × 10^−9^
Z_HER2:2891_-(S_3_G)_3_-ABD-mcDM1	HER2	7.1 × 10^4^	2.5 × 10^−4^	3.5 × 10^−9^
	HSA	8.5 × 10^4^	1.5 × 10^−4^	1.8 × 10^−9^
	MSA	3.1 × 10^5^	3.3 × 10^−3^	1.1 × 10^−8^
Z_HER2:2891_-(G_3_S)_3_-ABD-AA	HER2	1.2 × 10^6^	2.6 × 10^−4^	2.2 × 10^−10^
	HSA	7.9 × 10^5^	1.9 × 10^−4^	2.5 × 10^−10^
	MSA	6.3 × 10^6^	4.5 × 10^−3^	7.1 × 10^−10^
Z_HER2:2891_-(S_3_G)_3_-ABD-AA	HER2	8.8 × 10^5^	6.6 × 10^−4^	7.5 × 10^−10^
	HSA	9.8 × 10^5^	1.9 × 10^−4^	1.9 × 10^−10^
	MSA	4.5 × 10^6^	3.3 × 10^−3^	7.4 × 10^−10^
Z_HER2:2891_-G_4_S-ABD-mcDM1	HER2	7.5 × 10^5^	1.9 × 10^−4^	2.5 × 10^−10^
	HSA	8.0 × 10^5^	2.1 × 10^−4^	2.6 × 10^−10^
	MSA	3.8 × 10^6^	2.8 × 10^−3^	7.4 × 10^−10^

**Table 2:** In vitro cytotoxicity of Affibody-mcDM1 conjugates.

Cell Line	IC_50_ (nM)		
	Z_HER2:2891_-(G_3_S)_3_-ABD-mcDM1	Z_HER2:2891_-(S_3_G)_3_-ABD-mcDM1	Z_HER2:2891_-G_4_S-ABD-mcDM1
SKOV3	40	150	180
SKBR3	2.8	2.7	0.97
AU565	2.3	2.4	1.2

**Table 3:** Radiochemical yield, purity and stability of AffiDCs after 99mTc-labeling. The affibody-mcDM1 conjugates were incubated for 4 h with a 1000-fold molar excess of histidine and compared with the PBS control.

	Yield (%)	Purity (%)	Stability under 4 h Histidine Challenge (%)	
			Histidine	Control
^99m^Tc-Z_HER2:2891_-(G_3_S)_3_-ABD-mcDM1	95 ± 1 (n = 4)	99 ± 2 (n = 2)	98 ± 0 (n = 2)	98 ± 0 (n = 2)
^99m^Tc-Z_HER2:2891_-(S_3_G)_3_-ABD-mcDM1	93 ± 2 (n = 4)	98 ± 2 (n = 2)	98 ± 0 (n = 2)	98 ± 1 (n = 2)
^99m^Tc-Z_HER2:2891_-(G_4_S)-ABD-mcDM1	96 ± 1 (n = 6)	99 ± 1 (n = 2)	98 ± 0 (n = 2)	99 ± 0 (n = 2)

**Table 4:** Equilibrium dissociation constants (K_D_) for the interaction between ^99m^Tc-labeled affibody-mcDM1 conjugates with SKOV3 cells.

	K_D1_ (nM)	K_D2_ (nM)
Z_HER2:2891_-(G_3_S)_3_-ABD-mcDM1 (n = 2)	1.3 ± 0.4	22.5 ± 0.7
Z_HER2:2891_-(S_3_G)_3_-ABD-mcDM1 (n = 2)	1.0 ± 0.4	17.0 ± 3.6
Z_HER2:2891_-G_4_S-ABD-mcDM1 (n = 2)	0.56 ± 0.20	18.4 ± 2.9

**Table 5:** Comparative biodistribution of ^99m^Tc-labeled Z_HER2:2891_-(G_3_S)_3_-ABD-mcDM1, Z_HER2:2891_-(S_3_G)_3_-ABD-mcDM1, and Z_HER2:2891_-G_4_S-ABD-mcDM1 at 4, 24, and 48 h post injection (p.i.) in NMRI mice. The uptake is presented as % injected dose (ID)/g (average from 4 mice ± standard deviation (SD)). Data for intestines with content and carcass are presented as % ID per whole sample. Unpaired *t*-test was performed to find significant differences between the groups.

	-(G_3_S)_3_-			-(S_3_G)_3_-			-G_4_S-		
	4 h	24 h	48 h	4 h	24 h	48 h	4 h	24 h	48 h
**Blood**	11 ± 1	4.5 ± 0.6	2.7 ± 0.3 *^a,b^*	11 ± 2	3.9 ± 0.4	2.2 ± 0.2 *^a,c^*	11.1 ± 0.1	3.9 ± 0.6	1.8 ± 0.1 *^b,c^*
**Sal. gland**	1.9 ± 0.3	1.5 ± 0.1	1.0 ± 0.1 *^b^*	2.0 ± 0.4	1.3 ± 0.1	0.95 ± 0.05 *^c^*	2.3 ± 0.8	1.4 ± 0.3	0.82 ± 0.09 *^b,c^*
**Lung**	4.1 ± 0.8	2.1 ± 0.3	1.6 ± 0.1 *^b^*	4.2 ± 0.7	2.0 ± 0.2	1.4 ± 0.1 *^c^*	4.3 ± 0.7	2.0 ± 0.4	1.07 ± 0.04 *^b,c^*
**Liver**	4.0 ± 0.7 *^b^*	2.8 ± 0.3	2.4 ± 0.3	4.5 ± 0.8 *^c^*	2.7 ± 0.3	2.2 ± 0.2	5.8 ± 0.2 *^b,c^*	3.5 ± 0.7	2.2 ± 0.2
**Spleen**	2.1 ± 0.6	1.6 ± 0.3	1.6 ± 0.3 *^b^*	2.5 ± 0.4	1.3 ± 0.2	1.4 ± 0.2 *^c^*	2.5 ± 0.3	1.8 ± 0.5	1.1 ± 0.1 *^b,c^*
**Stomach**	1.5 ± 0.2 *^b^*	1.0 ± 0.2	0.65 ± 0.06 *^b^*	1.8 ± 0.3	0.9 ± 0.2	0.65 ± 0.08 *^c^*	2.2 ± 0.3 *^b^*	1.0 ± 0.2	0.54 ± 0.04 *^b,c^*
**Kidney**	89 ± 13	44 ± 5	20 ± 2	86 ± 15	36 ± 2	24 ± 2 *^c^*	94 ± 10	37 ± 8	19 ± 1 *^c^*
**Muscle**	0.73 ± 0.03	0.7 ± 0.1	0.48 ± 0.03 *^b^*	0.8 ± 0.2	0.6 ± 0.1	0.45 ± 0.04 *^c^*	0.8 ± 0.1	0.6 ± 0.1	0.36 ± 0.03 *^b,c^*
**Bone**	1.7 ± 0.2	1.5 ± 0.2	1.13 ± 0.04	1.8 ± 0.3	1.2 ± 0.1	1.18 ± 0.09 *^c^*	2.0 ± 0.4	1.5 ± 0.3	1.0 ± 0.1 *^c^*
**GI tract**	4.1 ± 0.7 *^a^*	3.0 ± 0.3	2.1 ± 0.5	10 ± 4 *^a^*	2.8 ± 0.4	1.9 ± 0.1	19 ± 13	3.0 ± 0.4	1.6 ± 0.3
**Carcass**	22 ± 3	18 ± 2	13 ± 1 *^b^*	22 ± 2	16 ± 2	13 ± 1 *^c^*	23 ± 3	17 ± 2	11 ± 1 *^b^*^,*c*^

*^a^* Significant difference between ^99m^Tc-labeled Z_HER2:2891_-(G_3_S)_3_-ABD-mcDM1 and Z_HER2:2891_-(S_3_G)_3_-ABD-mcDM1. *^b^* Significant difference between ^99m^Tc-labeled Z_HER2:2891_-(G_3_S)_3_-ABD-mcDM1 and Z_HER2:2891_-G_4_S-ABD-mcDM1. *^c^* Significant difference between ^99m^Tc-labeled Z_HER2:2891_-(S_3_G)_3_-ABD-mcDM1 and Z_HER2:2891_-G_4_S-ABD-mcDM1.

## Data Availability

The data generated during the current study are available from the corresponding author upon reasonable request.

## Supplementary Materials

The following supporting information can be downloaded at: https://www.mdpi.com/article/10.3390/pharmaceutics14030522/s1, Figure S1. Mass spectrometry data for affibody conjugates.
