# Supplementary material for: Effect of Inter-Domain Linker Composition on Biodistribution of ABD-Fused Affibody-Drug Conjugates Targeting HER2
Source: Pharmaceutics. 2022 Feb 26;14(3):522. doi: 10.3390/pharmaceutics14030522 (PMC8949183; doi:10.3390/pharmaceutics14030522)
Supplement: Supplementary file 1 [file pharmaceutics-14-00522-s001.zip › pharmaceutics-1586262-supplementary.pdf]

pharmaceutics

MDPI

Supplementary Materials: Effect of Inter-Domain Linker Composition on Biodistribution of ABD-Fused Affibody-Drug Conjugates Targeting HER2

Tianqi Xu, Jie Zhang, Maryam Oroujeni, Maria S. Tretyakova, Vitalina Bodenko, Mikhail V. Belousov, Anna Orlova, Vladimir Tolmachev, Anzhelika Vorobyeva and Torbjörn Gräslund

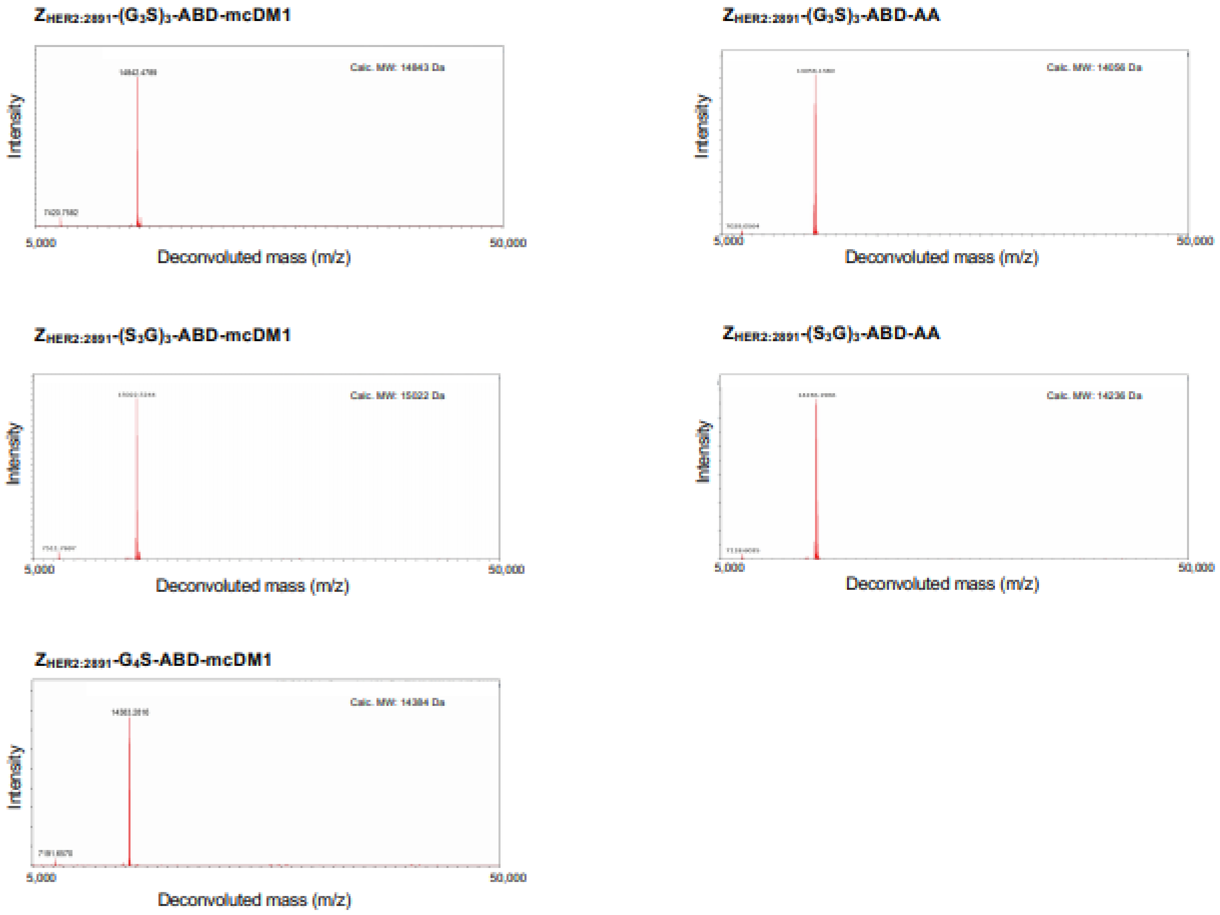

Figure S1. Mass spectrometry data for affibody conjugates.
